# Considerations for the Analysis of Small Extracellular Vesicles in Physical Exercise

**DOI:** 10.3389/fphys.2020.576150

**Published:** 2020-12-03

**Authors:** Alexandra Brahmer, Elmo W. I. Neuberger, Perikles Simon, Eva-Maria Krämer-Albers

**Affiliations:** ^1^Extracellular Vesicles Research Group, Institute of Developmental Biology and Neurobiology, Johannes Gutenberg University of Mainz, Mainz, Germany; ^2^Department of Sports Medicine, Rehabilitation and Disease Prevention, Johannes Gutenberg University of Mainz, Mainz, Germany

**Keywords:** physical exercise, extracellular vesicles, tissue cross-talk, circulation, plasma, EV isolation, standardization, exosomes

## Abstract

Physical exercise induces acute physiological changes leading to enhanced tissue cross-talk and a liberation of extracellular vesicles (EVs) into the circulation. EVs are cell-derived membranous entities which carry bioactive material, such as proteins and RNA species, and are important mediators of cell-cell-communication. Different types of physical exercise interventions trigger the release of diverse EV subpopulations, which are hypothesized to be involved in physiological adaptation processes leading to health benefits and longevity. Large EVs (“microvesicles” and “microparticles”) are studied frequently in the context of physical exercise using straight forward flow cytometry approaches. However, the analysis of small EVs (sEVs) including exosomes is hampered by the complex composition of blood, confounding the methodology of EV isolation and characterization. This mini review presents a concise overview of the current state of research on sEVs released upon physical exercise (ExerVs), highlighting the technical limits of ExerV analysis. The purity of EV preparations is highly influenced by the co-isolation of non-EV structures in the size range or density of EVs, such as lipoproteins and protein aggregates. Technical constraints associated with EV purification challenge the quantification of distinct ExerV populations, the identification of their cargo, and the investigation of their biological functions. Here, we offer recommendations for the isolation and characterization of ExerVs to minimize the effects of these drawbacks. Technological advances in the ExerV research field will improve understanding of the inter-cellular cross-talk induced by physical exercise leading to health benefits.

## Introduction

Strenuous physical exercise induces broad systemic changes in the body. In order to supply the tissues with an increasing amount of nutrients and oxygen, the respiratory, the cardiovascular and the neuromuscular systems are activated. Furthermore, enhanced shear forces, oxidative stress, and inflammatory reactions can be observed (Whyte and Laughlin, 2010; Schild et al., 2016). When performed regularly under moderate conditions, these bodily reactions lead to health benefits and disease prevention (Warburton and Bredin, 2017). Extracellular vesicles (EVs) are important players of cell-cell communication and are expected to contribute to these beneficial adaptations (Safdar and Tarnopolsky, 2018).

Extracellular vesicles compose of a phospholipid bilayer membrane encapsulating proteins, lipids, metabolites, and nucleic acid species, which differ depending on their parent cells, environmental factors, and stimuli (Colombo et al., 2014; van Niel et al., 2018). Three main types of EVs can be differentiated: exosomes originating from the endosomal machinery with a size of 30–100 nm, microvesicles in the size of 150–1,000 nm directly shedding from the plasma membrane, and apoptotic bodies formed as large vesicles during apoptosis. Upon diverse stimuli, EVs are released by most cell types into the interstitial fluid and body fluids, including blood, urine, lymph, and cerebrospinal fluid (Yáñez-Mó et al., 2015).

Extracellular vesicles in blood comprise a heterogeneous mixture of vesicles derived from platelets, red blood cells (together >50%), other circulating cells, and cells of the surrounding tissues (Arraud et al., 2014; Yáñez-Mó et al., 2015). Due to the lack of exclusive marker proteins and purification strategies for the EV-subclasses, the primary criteria to differentiate EVs in blood are size (Tkach et al., 2018). Thus, it became common to define EVs in the size of exosomes as small EVs (sEVs) and EVs above this size as large EVs. Cryo-electron microscopy analysis indicates an EV concentration of ~50,000/μl plasma, however, reports on EV numbers in blood are diverse (200–10^9^/μl plasma), highly depending on the method of examination (Shet et al., 2003; Dragovic et al., 2011; Arraud et al., 2014). Their presence in the circulation is restricted to few minutes or hours before they reach their targets (Takahashi et al., 2013; Matsumoto et al., 2020).

Next to the variety of circulating EVs, blood contains other bioactive components in submicron size, including plasma proteins, lipoproteins (Simonsen, 2017), and exomeres (Zhang et al., 2018). The heterogeneity of EV populations and other bioactive particles in blood faces EV research with numerous challenges and, thus, confuses the determination of EVs released into the circulation upon physical exercise (ExerVs). The modalities of ExerV-release, the putative role in adaptation signaling as well as prospective therapeutic and diagnostic applications were recently highlighted and comprehensively summarized (e.g., Trovato et al., 2019; Fuller et al., 2020; Vechetti et al., 2020). Here, we supplement this body of literature with a compilation of the most relevant technical limitations regarding ExerV isolation and characterization, focusing on sEVs. We provide a concise guideline for ExerV analysis and data interpretation, which we hope will help to further develop this young and promising research field.

## EVs in Physical Exercise

The different physiological stimuli during physical exercise lead to an alteration of the EV landscape in blood. Research in humans was mainly focused on flow cytometric analysis of large EVs from platelets and endothelial cells, also called microparticles. As reviewed in detail elsewhere (Eichner et al., 2018; Wilhelm et al., 2018), the concentration of platelet microparticles increases during physical activity, starting at an early phase of exercise and reaching baseline few hours after the exercise session. Their release has been attributed to the activation of coagulative processes (Ahmadizad et al., 2010) and shear stress (Wilhelm et al., 2016). In contrast, abundance of endothelial microparticles varied between studies, but was reported as unchanged in most cases after exercise (Eichner et al., 2018; Wilhelm et al., 2018).

Recently, sEVs have caught attention in the context of physical activity and an increasing number of studies addressed the release and their possible involvement in signaling pathways. Some studies in humans and rodents observed an immediate increase of sEVs after a single bout of physical exercise (Frühbeis et al., 2015; Bei et al., 2017; Oliveira et al., 2018; Whitham et al., 2018; Brahmer et al., 2019). One study found a direct reduction of total EV numbers, while detecting an increased population of muscle cell-derived EVs (Rigamonti et al., 2019). Furthermore, elevation of resting EV levels were detected in response to long-term exercise interventions (Chaturvedi et al., 2015; Bei et al., 2017; Bertoldi et al., 2018; Ma et al., 2018), though Hou et al. (2019) could not detect changes in EV levels. ExerVs appear as a complex mixture of vesicles originating from platelets (Frühbeis et al., 2015; Brahmer et al., 2019), endothelial progenitor, or endothelial cells (Ma et al., 2018; Brahmer et al., 2019; Hou et al., 2019), leukocytes (Brahmer et al., 2019), and muscle cells (Guescini et al., 2015; Whitham et al., 2018; Rigamonti et al., 2019), which most probably varies depending on exercise mode and time of investigation.

Analysis of the protein cargo of ExerVs identified various proteins associated with key signaling pathways, including angiogenesis, immune signaling, and glycolysis (Bryl-Górecka et al., 2018; Whitham et al., 2018; Brahmer et al., 2019; Just et al., 2020). Additionally, the secretion and transport of myokines *via* ExerVs was suggested (Whitham et al., 2018). Moreover, several studies found evidence for the transport of an altered miRNA panel *via* sEVs in response to exercise bouts or training (Chaturvedi et al., 2015; Guescini et al., 2015; D’souza et al., 2018; Lovett et al., 2018; Ma et al., 2018; Oliveira et al., 2018; Hou et al., 2019; Yin et al., 2019; Just et al., 2020). In acute exercise settings, this alteration was restored after 4 h or later (D’souza et al., 2018; Yin et al., 2019). Some of the miRNAs carried by ExerVs belong to the group of myomirs indicating involvement of EVs in muscle regeneration processes following exercise (Guescini et al., 2015; Yin et al., 2019). Functional analysis of ExerVs suggested contribution to cardio protection in ischemia/reperfusion-injury (Bei et al., 2017; Hou et al., 2019), hypoxia/reoxygenation-assays (Hou et al., 2019), tissue remodeling (Chaturvedi et al., 2015), endothelial function (Ma et al., 2018), as well as muscle remodeling and growth (Just et al., 2020) potentially mediated by ExerV-cargo transported in response to exercise stimuli.

Interestingly, no major discrepancies in the characteristics of ExerVs were detected between human, mouse, or rat models. Though, several aspects were identified which may influence specific results, including exercise setting (e.g., load or type of exercise; Frühbeis et al., 2015; Ma et al., 2018; Whitham et al., 2018; Brahmer et al., 2019; Yin et al., 2019), daytime (Bertoldi et al., 2018), age (Bertoldi et al., 2018), sex (Rigamonti et al., 2019), and body-mass-index (Rigamonti et al., 2019).

**Figure 1:**
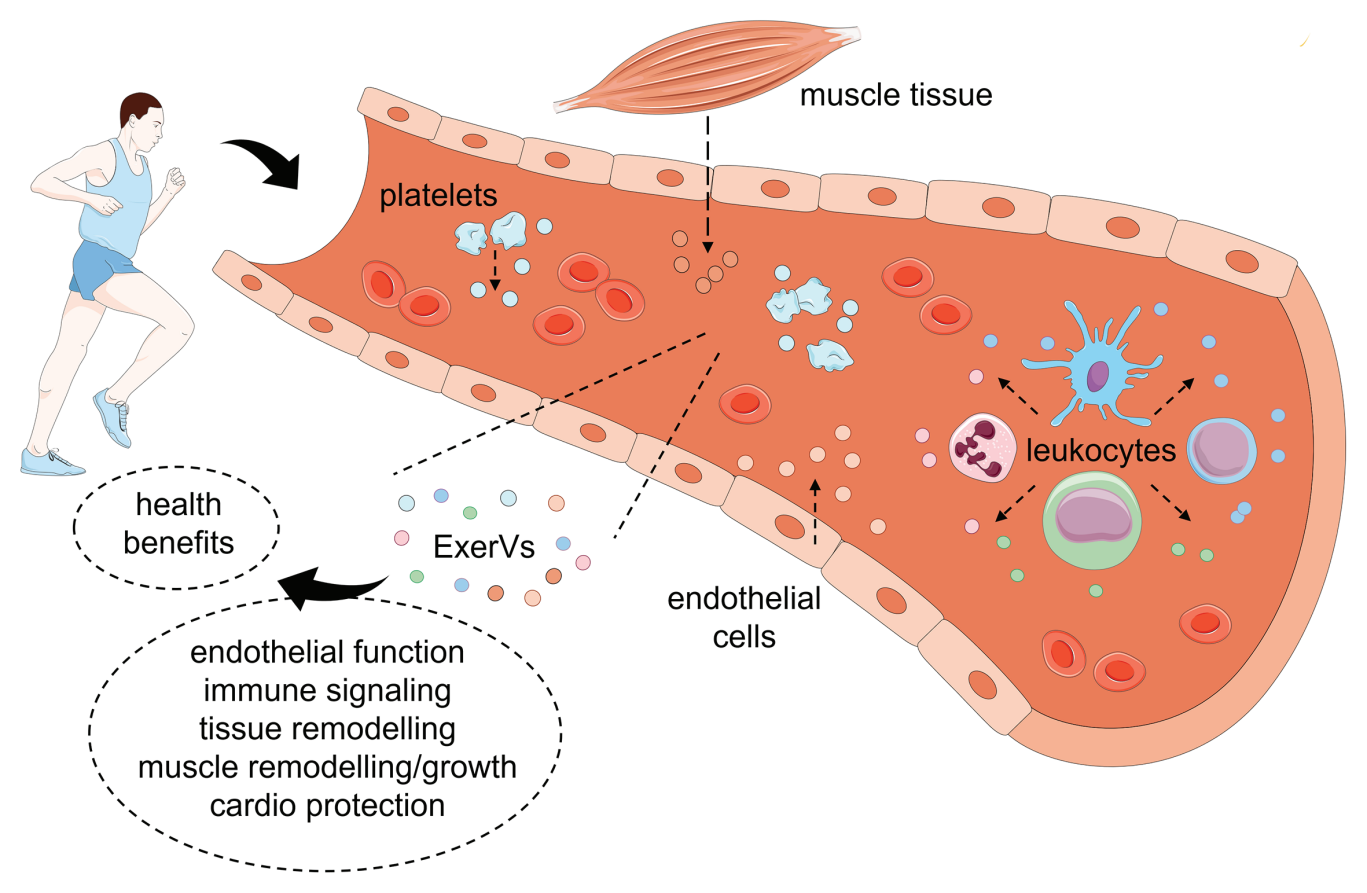
Model of extracellular vesicle (EV) release and function in response to physical exercise. During or after physical exercise, platelets, endothelial cells, leukocyte subsets, and muscle tissue release a complex mixture of EVs into the blood stream (ExerVs). These may deliver bioactive cargo in key signaling processes and mediate adaptational processes leading to health benefits. This figure was created using Servier Medical Art templates, which are licensed under a Creative Common Attribution 3.0 Generic License.

Overall, these studies provide evidence that sEVs are actively released into the circulation upon physical exercise and may function as mediators of different key signaling pathways, possibly involved in adaptation processes triggered by exercise (Figure 1). However, the awareness of potential flaws in the isolation and characterization of blood plasma EVs suggests a critical reflection of data interpretation regarding the side-effects caused by co-isolated non-EV components.

## Limitations of sEV Analysis in Blood Plasma

### Isolation

The common strategies to separate sEVs from blood plasma are highly susceptible to co-isolate lipoproteins, plasma proteins, including albumin, clotting factors, immunoglobulins, and other macromolecules likewise present in blood. Frequently used EV isolation techniques are differential ultracentrifugation (dUC; Théry et al., 2006) and commercial precipitation-based methods (e.g., “ExoQuick”; Van Deun et al., 2017). However, application of dUC or EV precipitation results in low-purity EVs with high amounts of co-isolated plasma proteins and lipoproteins and may promote the formation of aggregates (Linares et al., 2015; Lobb et al., 2015). Consequently, different EV purification techniques were developed aiming at separation of EVs from these main contaminants (for a detailed review, see Monguió-Tortajada et al., 2019). Lipoproteins, which are found in concentrations of 10^16^/ml plasma, share either size (chylomicrons, very low-density lipoprotein) or density (high-density lipoprotein) with EVs (Simonsen, 2017). Size exclusion chromatography (SEC) separates the majority of plasma proteins and small lipoprotein particles from EVs (Böing et al., 2014), which can be subjected to various downstream analysis methods. However, the remaining contaminants (large lipoprotein particles, among others) still hamper the subsequent use of SEC-EVs for sensitive downstream analysis like RNA-sequencing or proteome analysis and functional analyses. It turned out that a combination of different EV isolation strategies leads to high-purity EVs. Especially combination with density gradient centrifugation designed to separate EVs from large lipoproteins with lower density was successful in improving purity of EV preparations. For example, enrichment of EVs on a density cushion before purification with SEC led to reduced lipoprotein co-isolation and enabled detailed proteomic and RNA analysis (Karimi et al., 2018). Also, purity of sEVs was markedly increased by sequential dUC and density gradient centrifugation or immuno-affinity capture (Jeppesen et al., 2019). However, these approaches are highly laborious and associated with low recovery, hampering their application in clinical settings. Immuno-affinity isolation (e.g., using CD63-antibody coupled beads) including magnetic separation of captured EVs from plasma components offers a quick possibility to enrich for specific EV populations (Greening et al., 2015; Kowal et al., 2016; Nakai et al., 2016). However, applying immuno-affinity isolation for specific surface proteins introduces a selection bias for the chosen EV-associated protein and affinity beads are difficult to remove from EVs for intended functional analyses. Conclusively, a method for complete separation of EV subtypes from other plasma components is presently lacking and the technique should be carefully chosen based on the research aim and the subsequent EV analysis strategy.

### Characterization

Given the complex composition of the plasma-EV pool and the limitations in EV isolation, plasma-EV characterization is similarly challenging. Western blotting, nanoparticle tracking analysis (NTA), and electron microscopy are the most frequently used methods to characterize EV size and composition (Gardiner et al., 2016). Electron microscopy, in particular when coupled with immuno-gold labeling, is a valuable technique for EV characterization on single-EV level (Arraud et al., 2014; van der Pol et al., 2014). However, this technique is laborious and only available in expertized facilities. NTA offers a fast possibility to estimate a size profile and concentration of an EV sample (Dragovic et al., 2011). Though, this technique does not cover the complete size spectrum of EVs, and enumeration is highly influenced by co-isolates (Yuana et al., 2014; Jamaly et al., 2018). We noticed that intake of a high-fat meal had an enormous effect on particle numbers estimated by NTA, which were elevated by an order of magnitude under postprandial conditions indicating an increased co-isolation of lipoproteins (Brahmer et al., 2019). Similarly, flow cytometric analysis of large EVs is highly confounded by food-intake (Sódar et al., 2016). These findings indicate that EV enumeration using conventional NTA or flow cytometry (FC) is highly susceptible to lipoprotein contamination, which is dominating in absolute numbers even in samples derived from starved individuals, and thus cannot be consulted to determine EV-numbers or yield. While vesicle-specific labeling may improve accuracy, commonly used membrane dyes seem to fail in labeling all EVs in a sample or additionally stain non-EV sample components (de Rond et al., 2018). To circumvent these technical challenges, multiple innovative EV isolation methods, which include flow field-flow fractionation (Sitar et al., 2015) and acoustic approaches (Lee et al., 2015; Rezeli et al., 2016), were introduced. In addition, the invention of proper EV reference material has gained increasing attention (Valkonen et al., 2017; Geeurickx et al., 2019; Lozano-Andrés et al., 2019). These developments may provide the possibility to estimate actual ExerV concentrations and yield in future experiments.

### Dynamics and Phenotyping

Further information about the nature of the vesicles and potential EV-subpopulations is provided by the presence of the tetraspanins CD9, CD63, and CD81, other genuine EV markers such as TSG101, Syntenin, or Alix (Théry et al., 2018; Jeppesen et al., 2019) and cell type specific markers embedded in the membranes of the vesicles. These enable examination of EV level dynamics and the cell types of origin. Therefore, next to straight forward Western blotting or highly sensitive mass spectrometry proteome analysis, microarray, or FC techniques are frequently applied (Jørgensen et al., 2013; van der Pol et al., 2014; Arraud et al., 2016; Tian et al., 2018). For the latter, especially the resolution of conventional FC devices constitutes a main obstacle since particles smaller than 500 nm are difficult to detect (Erdbrügger et al., 2014; Welsh et al., 2020). Technical improvements in FC sensitivity, the use of strategic fluorescence labeling in FC (e.g., immunobead-based multiplexed assay, Koliha et al., 2016), and combination of FC with high-resolution imaging (Lannigan and Erdbruegger, 2017; Görgens et al., 2019) continuously advance the EV-phenotyping technology. Still, availability of a robust high-throughput single-EV detection method would be crucial to determine the full dynamics and phenotypes of defined EV subpopulations in the complex pool of ExerVs.

### Cargo

The analysis of proteomic and nucleic acid content in plasma-EVs by mass-spectrometric profiling or RNA-sequencing, respectively, is important to reveal functional properties of EVs. As introduced above, the choice of isolation technique may massively confound omics results (Van Deun et al., 2014; Simonsen, 2017). For example, lipoproteins are also capable of transporting RNA species (Vickers et al., 2011) and miRNA mediated results can easily be misinterpreted as EV-specific while being a result of lipoprotein co-isolation.

In conclusion, plasma-EV and thus ExerV isolation and characterization are highly demanding tasks. One must carefully consider the combination of EV purification and subsequent characterization method in order to prevent contaminants falsifying the experimental outcomes. Existing technical limits need to be considered for accurate data interpretation.

## Discussion

### Rigorous ExerV Analysis

**Figure 2:**
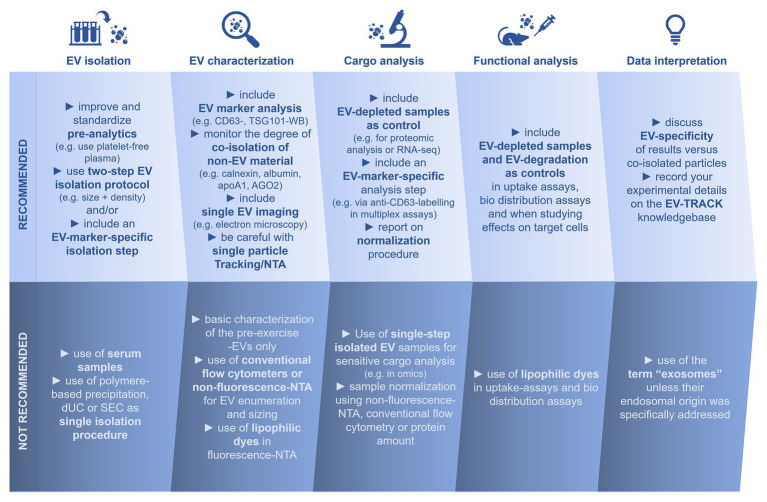
Considerations for the analysis of extracellular vesicles in physical exercise. *EV*, extracellular vesicle; *dUC*, differential ultracentrifugation; *SEC*, size exclusion chromatography; *NTA*, nanoparticle tracking analysis; *CD*, cluster of differentiation; *TSG101*, Tumor susceptibility gene 101; *WB*, Western blot analysis; *apoA1*, apolipoprotein1; *AGO2*, argonaute2 protein; and *RNA-seq*, RNA-sequencing.

The present reports on sEVs or exosome-like EVs in exercise settings comprise a collection of multiple different EV isolation methods and characterization strategies. Diverse separation methods were used to study the amount, cargo and functions of ExerVs from human, mouse, and rat plasma or serum: centrifugation at 20,000 × *g* (Whitham et al., 2018), dUC (Chaturvedi et al., 2015; Frühbeis et al., 2015; Guescini et al., 2015; Ma et al., 2018; Hou et al., 2019; Rigamonti et al., 2019), chemical precipitation (Bei et al., 2017; Bertoldi et al., 2018; Oliveira et al., 2018; Hou et al., 2019; Yin et al., 2019; Just et al., 2020), SEC (D’souza et al., 2018; Lovett et al., 2018; Brahmer et al., 2019; Just et al., 2020), immuno-affinity capture (Guescini et al., 2015; Ma et al., 2018; Brahmer et al., 2019), and acoustic trapping (Bryl-Górecka et al., 2018). Importantly, these studies consistently reported increasing amounts of EVs during or after acute or chronic exercise, changes in the miRNA and proteomic cargo of ExerVs as well as beneficial effects of ExerVs in key signaling pathways. However, these observations should be reflected regarding a potential co-isolation of macromolecular complexes (a) influencing particle enumeration, (b) contributing to RNA and proteins mistakenly designated as ExerV cargo, and (c) responsible for effects observed in functional analysis. Following the MISEV recommendations of the International Society for Extracellular Vesicles (Théry et al., 2018), we extrapolate this advice for ExerV analysis and address the most striking technical hindrances which could impact ExerV study interpretation (Figure 2).

A solid basis for ExerV analysis is provided by careful selection of the preanalytical factors (Lacroix et al., 2012; Théry et al., 2018). Standardized sample processing including the preparation of platelet-free plasma (Lacroix et al., 2012) will reduce influence of preanalytical factors on ExerV analysis. For the separation of ExerVs from plasma, the use of a two-step isolation protocol (e.g., isolating for size and density, subsequently) or inclusion of an EV-marker specific isolation step (e.g., CD63-immuno-affinity capture) is beneficial. In interest of reproducibility, the isolation procedure (centrifugal forces, pore sizes, used kits, etc.) as well as sample storage conditions (buffer, temperature, etc.) should be comprehensively reported. One should consider that a freezing cycle (of blood plasma or isolated vesicles) can lead to changes in vesicle properties (Lorincz et al., 2014; Yuana et al., 2015) and that initial investigations should better be performed with freshly obtained sample material and confirmed using frozen samples. A full basic ExerV characterization includes biochemical EV-marker analysis, single-EV imaging and single particle tracking (further details can be found in the MISEV-guidelines; Théry et al., 2018). Consequent monitoring of the degree of co-isolation of non-EV material, e.g., *via* Western blotting for non-EV markers like apolipoproteins, plasma proteins, and RNA-binding proteins, offers improved evaluation of study results. Since exercise triggers many systemic changes, it is not sufficient to characterize pre-exercise EVs in detail and assume similar basic characteristics, including purity, of the isolated EV material under exercise conditions. Similarly, a modification of the EV isolation technique requires updated EV characterization. Importantly, we do not recommend relying on non-fluorescence-NTA and conventional FC for EV enumeration and subsequent normalization in downstream experiments. The results will be misleading, due to a strong bias introduced by remaining lipoproteins. In fluorescence-NTA, antibody labeling of EVs may be preferred over the use of lipophilic dyes. Likewise, normalization according to total protein is not recommended, given the high degree of plasma protein co-isolation. It should be noted, that most presently available methods of EV-quantification operate at a semi-quantitative level.

To avoid confounders and misinterpretation of proteomic and transcriptomic data as well as biodistribution and functional analysis, it may be helpful to include control conditions utilizing EV-depletion or EV-degradation. These controls may indicate, although not absolutely confirm, EV-specificity of the results. Discussion of the latter is recommended for all experimental details. Notably, the term sEVs may be preferred over the term exosomes, unless the endosomal origin of EVs has been verified (Witwer and Théry, 2019). We propose using the term ExerVs to include small and large EV-subpopulations into analysis and study interpretation.

A valuable platform to report on experimental details and get technical information about published work on EVs and ExerVs is the EV-TRACK knowledgebase (Van Deun et al., 2017). On a long-term, optimized ExerV sample preparation and characterization as well as transparent reporting will lead to reliable results and improve inter-study comparison in the future.

## Future

The involvement of ExerVs in health-promoting adaptation processes initiated by physical exercise suggested in the current literature represents a valid and attractive working hypothesis. In future studies, a well-designed strategy of improved EV isolation involving sequential purification steps that reduce co-isolation of non-EV material is instrumental to overcome the methodical challenges of ExerV characterization. Downstream examination of high-purity ExerVs will provide a more precise picture of the proteomic, metabolomic, and nucleic acid content of defined ExerV subpopulations and their multiple functions. Moreover, standardized EV-methodology is required to implement high-throughput analysis of ExerVs. We encourage ExerV researchers to make use of community-driven tools provided by the International Society of Extracellular Vesicles, like the EV-TRACK knowledgebase, and to follow the regularly updated MISEV-guidelines. The EV research field is developing rapidly, and improved purification and analysis techniques will be instrumental to unravel the role of EVs in the adaptation processes in response to exercise in the future.

## Author Contributions

AB conceived and wrote the manuscript. EN, PS, and E-MK-A critically discussed the content and revised the manuscript. All authors contributed to the article and approved the submitted version.

### Conflict of Interest

The authors declare that the research was conducted in the absence of any commercial or financial relationships that could be construed as a potential conflict of interest.
